# Fibrillatory wave amplitude and thromboembolic risk in non-anticoagulated patients with atrial fibrillation

**DOI:** 10.1080/07853890.2024.2317362

**Published:** 2024-02-13

**Authors:** Arto Relander, Samuli Jaakkola, Hilla Virri, Eelis Niemelä, Tuija Vasankari, Ilpo Nuotio, K. E. Juhani Airaksinen, Tuomas Kiviniemi

**Affiliations:** a Heart Center, Turku University Hospital and University of Turku, Turku, Finland; b Department of Medicine, Turku University Hospital and University of Turku, Turku, Finland

**Keywords:** Atrial fibrillation, electrocardiogram, F-wave, stroke

## Abstract

**Background:**

The benefit of oral anticoagulation in atrial fibrillation (AF) is well established for patients at elevated stroke risk, but less clear for those at intermediate risk. We investigated whether analysis of electrocardiogram (ECG) derived fibrillatory waves (F-waves) could help identify patients at risk for stroke and systemic embolism (SSE).

**Methods:**

The Finnish Cardioversion (FinCV) study included patients not on permanent anticoagulation therapy who underwent cardioversion for an acute AF episode. We identified 739 individuals with a valid ECG and complete follow-up data. The maximum amplitudes of the F-waves in leads II and V1 were manually measured from the pre-procedure ECG. Patients were categorized into fine and coarse F-wave groups. The optimal lead and amplitude threshold for grouping were found in an events per person-years analysis. SSE were identified from the patient medical records until either anticoagulation was prescribed, AF was deemed chronic, the patient had deceased, or the end of follow-up.

**Results:**

Overall 37 (5.0%) patients suffered SSE during the median follow-up time of 5.4 years (1.9–10.8). Measured from lead V1 the SSE rates per 100 person-years were 1.5 and 0.7 in fine and coarse F-wave groups, respectively. Fine F-waves were observed in 112 (15.2%). Baseline characteristics were similar between the groups. Fine F-wave predicted SSE in a competing risk analysis (SHR 2.34, 95%CI 1.12–4.87, *p* = .023). Analyses from lead II did not provide significant results.

**Conclusion:**

Electrocardiographic F-wave amplitude may provide additional information on stroke risk in patients with paroxysmal AF and borderline indications or contraindications for anticoagulation.

## Introduction

Fibrillatory waves (F-waves) in the electrocardiogram (ECG) may indicate abnormal atrial substrate in patients with atrial fibrillation (AF). Instead of representing disorganized and chaotic contractility, F-wave amplitudes are consistent between ECG recordings over time [1], and larger amplitudes reflect more regular atrial activity [2]. However, the clinical significance of F-wave amplitude remains unclear.

The F-wave amplitude seems to be negatively associated with increasing age and AF duration, suggesting that viable myocytes might be replaced by fibrosis [3–7]. Additional stroke risk factors, such as underlying cardiac disease or left atrial appendage dysfunction, do not appear to be clearly related to F-wave amplitude [5–11]. It should be noted that most of these studies categorize patients into fine and coarse F-wave groups utilizing an amplitude cut-point of 1.0 mm, which was first proposed by Peter et al. solely for being more convenient to measure [8]. In this study, we sought to re-evaluate the optimal F-wave cutoff point and explore its significance in predicting stroke and systemic embolisms (SSE) over time in non-anticoagulated AF patients.

## Materials and methods

**Figure 1.**
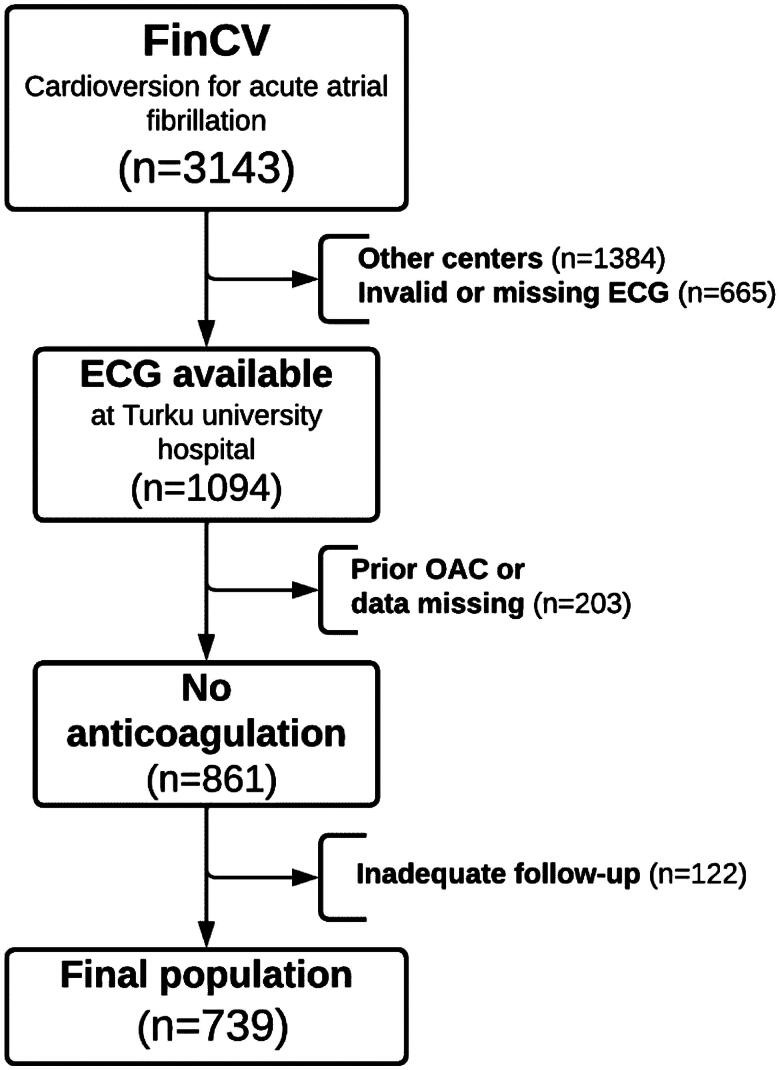
Flow chart presenting study inclusion criteria. ECG: electrocardiogram; FinCV: Finnish cardioversion study; OAC: oral anticoagulant.

The Finnish Cardioversion (FinCV) study (http://www.clinicaltrials.gov, identifier NCT04001205) is part of a wider protocol in progress to assess clinical challenges of AF in Finland [12,13]. The original patient cohort for this study consisted of individuals (>18 years of age) who underwent a cardioversion (CV) for acute (<48 h) AF. The discharge records of the three participating hospitals were retrospectively reviewed using the ICD-code for AF (I48) and NCSP procedure code for CV (TFP20) to identify the patients. Furthermore, only patients permanently living in the hospital catchment area were qualified to ensure complete follow-up data. To be included in this sub-study, only patients with no peri-procedural or post-CV anticoagulation were selected, and patient ECGs had to be electronically available at Turku University Hospital *via* the MUSE-ECG database. A flowchart of the study setting is shown in Figure 1.

A structured case report form was used for baseline and follow-up data collection. Patients were followed-up after the index CV (2003–2010) until either death, oral anticoagulant prescription, AF was deemed chronic, or follow-up ended (2019–2020). The cause of death data were acquired from the National Death Registry, Statistics Finland. The main outcome of interest was the composite of SSE, including fatal stroke.

### Electrocardiogram

Measurements were made manually from electronically stored ECGs by three observers blinded to the clinical data and study outcomes. A standard 12-lead ECG with 50 mm/s recording speed and 10 mm/mV voltage gain was acquired preceding the index CV. The precision for measurements was set to 0.25 mm and borderline figures were always rounded down.

**Figure 2.**
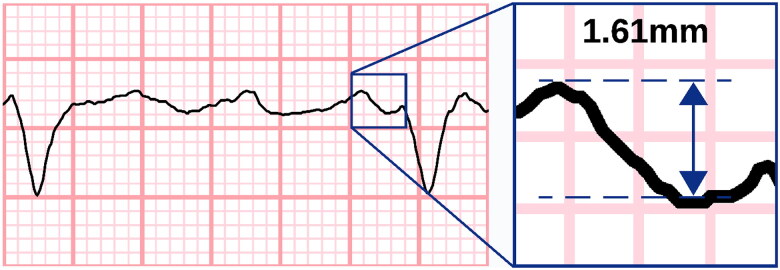
Measuring the fibrillatory wave amplitude. Fibrillatory waves were measured at the oscillating baseline not including QT intervals. Here, measurement was made from peak to trough; however, a trough-to-peak method was also accepted if it provided higher amplitudes. Electrocardiogram line thickness was set at 0.15mm. A coarse fibrillatory wave is presented with maximum amplitude of 1.61mm (measured with electronic callipers for demonstrative purposes). Measurements were rounded down to nearest 0.25mm. ECG with 50 mm/s recording speed and 10 mm/mV voltage gain.

The highest F-waves were visually identified in both leads II and V1. The F-wave amplitude was measured from peak-to-trough or trough-to-peak (Figure 2, Supplementary Figure 1) as described by Peter et al. [8] To mitigate any cumulative errors in amplitude, we restrained from assessing F-waves during the QT interval, and because of this restriction, ECGs with too high a heart rate had to be discarded. Recordings with insufficient general quality or atrial flutter instead of AF were also excluded from the study.

For further analysis, patients were categorized into two groups according to the maximum F-wave amplitude. Generally, amplitudes of ≥1 mm have been dubbed coarse F-waves and the rest fine F-waves. We utilized an events per person years analysis to find the amplitude cutoff point with the highest SSE event ratio for the most effective patient classification.

### Statistical analyses

Statistical analyses were conducted using SPSS statistical software (version 25.0; SPSS, IBM SPSS Inc., Chicago, IL, USA) and STATA version 16.1 (Stata Statistical Software, College Station, TX, StataCorp LLC). Continuous variables were not normally distributed and were therefore reported as medians (25th–75th percentiles). Categorical variables were described as counts (percentages). Pearson’s chi-square and Wilcoxon rank sum tests were used for univariate analyses, as appropriate. The relationships between continuous variables were studied with Spearman correlation. Fine-Gray competing risk analysis was used to assess the SSE risk over time in fine and coarse F-wave groups. The competing risk model was adjusted for sex and age. A *p*-value <.05 was considered statistically significant.

### Data quality

In addition to the maximum amplitudes, the second-highest F-waves were also measured from both leads V1 and II to control for intraobserver variability and to rule out abnormally high amplitudes caused by aberrations. Intraobserver correlations varied between *r* = 0.81–0.85 for HV (*n* = 239, *p* < .001), *r* = 0.81–0.86 for EN (*n* = 243, *p* < .001) and *r* = 0.78–0.80 for MM (*n* = 257, *p* < .001).

For interobserver variability an additional sample of 100 ECGs was analyzed by all observers. Interobserver correlations varied between *r* = 0.76–0.94 (*p* < .001).

A subgroup of 25 patients had two consecutive (within 30 d) ECGs analyzed to address the potential daily variability of F-wave amplitudes. The correlation between successive measurements was *r* = 0.84 (*p* < .001).

## Results

First, we sought to assess the best threshold for discriminating the SSE risk. There was a moderate correlation between the maximum F-wave amplitudes of leads II and V1 (*r* = 0.36, *p* < .001). SSE event rate/100 person years was analysed using 0.5, 0.75, 1.0 and 1.25 mm cut offs in both leads. The fine to coarse ratio of SSE risk varied from 1.0 to 2.2 in lead V1 and from 0.7 to 1.3 in lead II. The ratio was highest (2.2) for 0.5 mm cut off in lead V1 (Supplementary Figure 2). Based on this analysis, we divided the patients into two groups using the F-wave amplitude in lead V1. F-waves <0.5 mm in amplitude were considered fine and ≥0.5 mm were defined coarse F-waves. A total of 112 (15.2%) patients had fine F-waves in their baseline ECG.

In the entire cohort, the median age was 62 years (53–70), and 461 (62.4%) patients were female. The CHA_2_DS_2_-VASc score was <2 in 307 (58.5%) cases. The baseline characteristics of the patients with fine or coarse F-waves were comparable at the time of initial CV (Table 1), but at the end of follow-up, patients with fine F-waves were more likely hypertensive (*p* = .031) and had more often CHA_2_DS_2_-VASc scores ≥2 (*p* = .031).

**Table 1.** Clinical characteristics of patients with fine or coarse fibrillatory waves at the time of initial cardioversion and at the end of the follow-up.

	Fine (*n* = 112)	Coarse (*n* = 627)	*P*
At baseline			
Age, years	62 (56–70)	62 (53–70)	.408
Female sex	67 (59.8)	394 (62.8)	.597
Hypertension	55 (49.1)	263 (41.9)	.178
Heart failure	4 (3.6)	23 (3.7)	.999
Diabetes	11 (9.8)	54 (8.6)	.717
Vascular disease	16 (14.3)	106 (16.9)	.581
Prior stroke or TIA	6 (5.4)	26 (4.1)	.411
CHA_2_DS_2_-VASc	1 (0–3)	1 (0–3)	.581
≥2	49 (43.8)	258 (41.1)	.605
At the end of follow-up			
Follow-up duration, years	6.4 (2.1–10.0)	5.0 (1.7–9.8)	.355
Hypertension	71 (63.4)	326 (52.0)	.031
Heart failure	3 (2.7)	27 (4.3)	.604
Diabetes	19 (17.0)	91 (14.5)	.475
Vascular disease	18 (16.1)	113 (18.0)	.688
New thromboembolism	11 (9.8)	26 (4.1)	.018
CHA_2_DS_2_-VASc	3 (1–3)	2 (1–4)	.101
≥2	83 (74.1)	396 (63.2)	.031

Data are presented as median (interquartile range) for continuous variables and frequency (percentage) for categorical variables.

CHA_2_DS_2_-VASc: congestive heart failure, hypertension, age ≥75 (doubled), diabetes mellitus, and prior stroke, transient ischemic attack or thromboembolism (doubled), vascular disease, age 65–74, sex category (female); TIA: transient ischemic attack.

**Figure 3.**
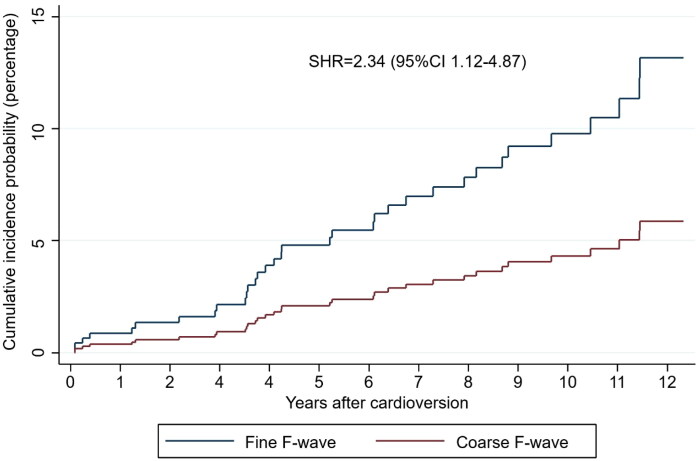
The cumulative incidence probability for strokes and systemic embolisms. Patients with a fine fibrillatory wave were more likely to experience stroke and systemic embolisms after index cardioversion. The competing risk model was adjusted for age and sex, and death from other causes was the competing risk. CI: confidence interval; F-wave: fibrillatory wave; SHR: subdistribution hazard ratio.

During the median follow-up period of 5.4 years (1.9-10.8), there were 37 (5.0%) SSEs. In the univariate analyses, the fine F-wave was associated with SSE (*p* = 0.018) (Table 1). Patients with SSE were older and had higher CHA_2_DS_2_-VASc scores (Table 2). In a competing risk analysis, a fine F-wave predicted SSE when death was the competing risk (SHR 2.34, 95%CI 1.12–4.87, *p* = .023), see Figure 3. The analysis was adjusted for age (per year [SHR 1.05, 95%CI 1.03–1.08, *p* < .001]) and female sex (SHR 1.32, 95%CI 0.64–2.72, *p* = .444).

**Table 2.** Characteristics for patients with or without incident stroke or systemic embolisms.

	Yes (*n* = 37)	No (*n* = 702)	*p*
At baseline			
Age, years	68 (61–73)	61 (53–70)	<.001
Female sex	23 (62.2)	438 (62.4)	.999
Hypertension	21 (56.8)	297 (42.3)	.090
Heart failure	2 (5.4)	25 (3.6)	.640
Diabetes	5 (13.5)	60 (8.5)	.363
Vascular disease	6 (16.2)	116 (16.5)	.999
Prior stroke or TIA	5 (13.5)	27 (3.8)	.018
CHA_2_DS_2_-VASc	2 (1–3)	1 (0–3)	<.001
≥2	26 (70.3)	281 (40.0)	<.001
F-wave amplitude	0.75 (0.25–0.75)	0.75 (0.50–1.0)	.153
<1.0 mm	30 (81.1)	488 (69.5)	.145
<0.5 mm	11 (29.7)	101 (14.4)	.018
At the end of follow-up			
Follow-up duration, years	6.1 (3.5–8.8)	5.1 (1.7–9.9)	.654
Hypertension	26 (70.3)	371 (52.8)	.043
Heart failure	1 (2.7)	29 (4.1)	.999
Diabetes	7 (18.9)	103 (14.7)	.477
Vascular disease	7 (18.9)	124 (17.7)	.826
CHA_2_DS_2_-VASc	5 (4–5)	2 (1–3)	<.001
≥2	37 (100)	442 (63.0)	<.001

Data are presented as median (interquartile range) for continuous variables and frequency (percentage) for categorical variables.

CHA_2_DS_2_-VASc: congestive heart failure, hypertension, age ≥75 (doubled), diabetes mellitus, and prior stroke, transient ischemic attack or thromboembolism (doubled), vascular disease, age 65–74, sex category (female); F-wave: fibrillatory wave; TIA: transient ischemic attack.

When the amplitude cutoff point of 1.0 mm was used in lead V1, the majority of patients were classified into the fine F-wave group (*n* = 518 [70.1%]), and no significant association with SSE was found (Supplementary Table 1). The analysis of F-wave amplitudes in lead II did not provide significant predictive power (data not shown).

## Discussion

This study examined the optimal cutoff point for categorizing patients by F-wave amplitude and its significance in predicting SSE in non-anticoagulated AF patients. A fine F-wave of less than 0.5 mm in amplitude was found in nearly one in six patients, 10% of whom suffered from an SSE. The risk of SSE was more than twice greater compared to those with coarse F-waves. The easily measured F-wave amplitude could aid clinical decision making when considering anticoagulation for a patient with otherwise borderline indications.

Our study utilized the unique FinCV database that was collected at the time when patients undergoing CV for AF were not routinely anticoagulated [12,13]. The strengths of using the FinCV database for assessing ECG-based risk include a larger sample size, longer follow-up, and inclusion of only non-anticoagulated patients compared to prior studies [5–7,11,14]. Previously, small studies with short follow-up periods have investigated F-waves and the occurrence of SSE with conflicting results [5–7,11,14]. Our results are in line with Yamamoto et al. [5], who found that fine F-waves (<1.0 mm) predicted SSE. However, the study was limited by the small size (*n* = 88) and the fact that only a fraction of patients was not using anticoagulation. In a larger study (*n* = 455) by Blackshear et al. [14] no association was found, but the follow-up was relatively short (median 1.3 years).

The underlying mechanisms causing changes in the F-wave amplitude are not well known. Large amplitudes in the ECG are generally associated with hypertrophy and chamber enlargement, whereas smaller voltages usually indicate fibrosis. Nevertheless, prior studies have found atrial enlargement in both fine [5] and coarse [7,15,16] F-wave groups, but mostly results have been inconclusive [10,11,14,17–19]. Atrial fibrosis has been suggested to affect F-wave amplitude reduction regardless of the left atrial diameter [20]. Fine F-waves have been previously associated with older age and a longer AF history, which could indicate fibrosis [3–7,21]. In our cohort, the groups were of similar age. Furthermore, fine F-waves have been associated with fragmented activation patterns and conduction delays [2]. There is no clear association of the left atrial appendage dysfunction with F-wave amplitude [5,6,10,11,14]. Thurmann et al. [15] discovered that all (*n* = 16) patients with autopsy-verified atrial enlargement had coarse (≥0.5 mm) F-waves; however, other information on histological properties was lacking.

We searched for an optimal cutoff point for SSE risk prediction because of inconclusive prior reports. We measured the amplitudes with 0.25 mm precision and analysed all possible variations for groupings. Previously, a threshold of 0.5 mm between fine and coarse F-waves was suggested to differentiate rheumatic AF from other etiologies [15] whereas cut-point of 1.0 mm correlated with pathological P-terminal force [8]. Investigations have shown that F-wave measurements are repeatable over time, which suggests a structural origin causing electromechanical changes [9,15,22]. In our cohort, a small subset of patients had consecutive ECGs analyzed to verify repeatability of measurements within 30 days.

### Limitations

This study has the inherent limitations of a registry-based study. ECG data were collected at the initial CV, after which patients were followed without regular study visits. Another important limitation is that no data on body mass index were available. Obesity could have a mitigating effect on F-wave amplitudes, especially in chest leads, and is a risk factor for SSE. Our study included only non-anticoagulated patients from years 2003 to 2010. At that time, the significance of the CHA_2_DS_2_-VASc score had not yet been established, and SSE risk stratification was based on physicians’ discretion; therefore, even high-risk patients were included. Finally, causal relationships could not be formally addressed by using a retrospective design.

## Conclusions

We demonstrated that a fine F-wave amplitude was correlated with an elevated risk of SSE. F-wave amplitude is an easily measured parameter that could help in risk stratification of borderline cases when considering oral anticoagulation. These results along with the underlying structural and histological mechanisms require further investigation.

## Supplementary Material

Supplemental Material

## Data Availability

The data underlying this article are available upon reasonable request from the corresponding author. Authors AR and TK had full access to the study data and take responsibility for its integrity and data analysis.
