# Supplementary material for: Fibrillatory wave amplitude and thromboembolic risk in non-anticoagulated patients with atrial fibrillation
Source: Ann Med. 2024 Feb 13;56(1):2317362. doi: 10.1080/07853890.2024.2317362 (PMC10866044; doi:10.1080/07853890.2024.2317362)
Supplement: Supplemental Material [file IANN_A_2317362_SM3977.docx]

**
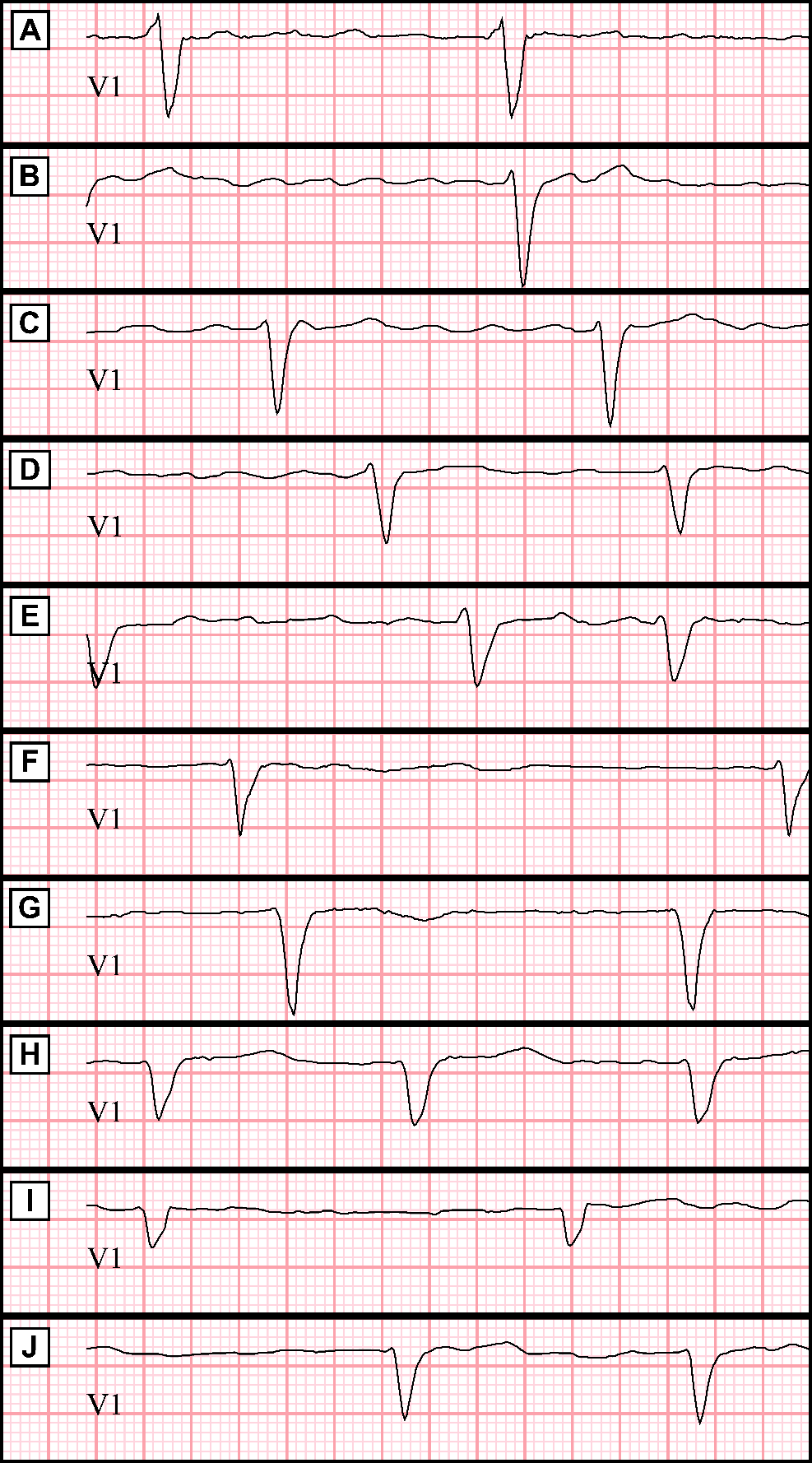
**

**
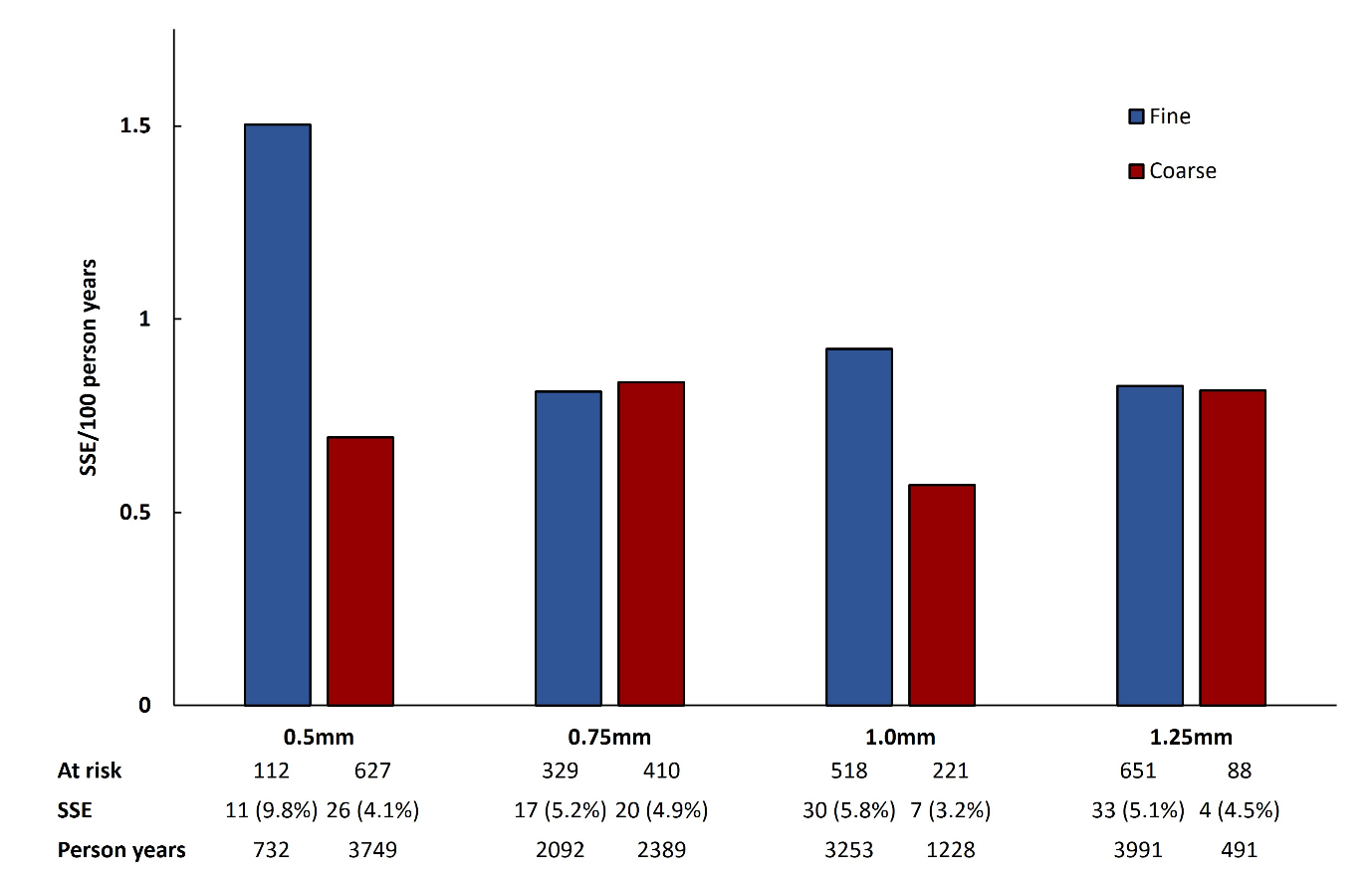
**

**Supplementary Figure S1 – Samples of coarse and fine F-waves**

Exact measurements were made with callipers for demonstrative purposes and further rounded down to nearest 0.25mm. Measurements during QT-interval were discarded. Images are samples of standard ECGs with 50 mm/s speed and 10 mm/mV voltage gain.

A: Coarse F-wave, maximum amplitude 0.86mm. B: A coarse F-wave, maximum amplitude 0.66mm. C: A coarse F-wave, maximum amplitude 0.71mm. D: A coarse F-wave, maximum amplitude 0.76mm. E: A coarse F-wave, maximum amplitude 0.85mm. F: A fine F-wave, maximum amplitude 0.46mm. G: A fine F-wave, maximum amplitude 0.46mm.H: A fine F-wave, maximum amplitude 0.31mm. I: A coarse F-wave, maximum amplitude 0.50mm. J: A coarse F-wave, maximum amplitude 0.55mm.

**Supplementary Figure S2 – Events per 100 person years for each amplitude cut off in lead V1.**

Event rates were calculated for both fine and coarse groups at each cut point. A clear difference can be observed at 0.5mm cut point, whereas rates at other cut points are nearly equal.

*SSE = stroke and systemic embolisms*

**Supplementary Table S1 – Clinical characteristics for fine and coarse fibrillatory wave groups at 1.0mm cut-off.**

|  | **Fine**  **(n=518)** | **Coarse**  **(n=187)** | **p** |
| --- | --- | --- | --- |
| At baseline |  |  |  |
| **Age, years** | 63 (54–71) | 60 (51–68) | 0.027 |
| **Female sex** | 320 (61.8) | 141 (63.8) | 0.620 |
| **Hypertension** | 237 (45.8) | 81 (36.7) | 0.023 |
| **Heart failure** | 17 (3.3) | 10 (4.5) | 0.399 |
| **Diabetes** | 46 (8.9) | 19 (8.6) | 0.999 |
| **Vascular disease** | 88 (17.0) | 34 (15.4) | 0.665 |
| **Prior stroke or TIA** | 24 (4.6) | 8 (3.6) | 0.693 |
| **CHA_2_DS_2_-VASc** | 1 (0–3) | 1 (0–3) | 0.032 |
| **≥2** | 225 (43.4) | 82 (37.1) | 0.121 |
| At follow-up |  |  |  |
| **Follow-up duration, years** | 5.5 (2.0–9.9) | 4.5 (1.4–9.6) | 0.088 |
| **Hypertension** | 290 (56.0) | 107 (48.4) | 0.064 |
| **Heart failure** | 18 (3.5) | 12 (5.4) | 0.226 |
| **Diabetes** | 83 (16.0) | 27 (12.2) | 0.214 |
| **Vascular disease** | 97 (18.7) | 34 (15.4) | 0.294 |
| **New thromboembolism** | 30 (5.8) | 7 (3.2) | 0.145 |
| **CHA_2_DS_2_-VASc** | 2 (1–4) | 2 (1–3) | 0.003 |
| **≥2** | 354 (68.3) | 125 (56.6) | 0.002 |

Data are given as median (interquartile range) for continuous variables and as frequency (percentage) for categorical variables.

*CHA_2_DS_2_-VASc = congestive heart failure, hypertension, age ≥75 (doubled), diabetes mellitus, and prior stroke, transient ischemic attack or thromboembolism (doubled), vascular disease, age 65 to 74, sex category (female); F-wave = fibrillatory wave; TIA = transient ischaemic attack*
